# The Electro-Chemical Treatment of Seed Grain
*Part of a paper read before the Agricultural Section of the British Association, September 9.


**Published:** 1919-10-11

**Authors:** A. E. Blackburn


### THE ELECTRO-CHEMICAL TREATMENT OF SEED GRAIN.*

By Dr. A. E. BLACKBURN.

ALL living matter—that of human beings, animal and plant life—is made up of solutions separated by walls and membranes. Those walls are permeable in different degrees. Continual changes are taking place in these solutions. To understand these changes is to understand life. The force which determines the movements of fluids in plants is called the osmotic pressure. Now, solutions which conduct the electric current, as a solution of salt—so called electrolytes—have a greater osmotic pressure than we should expect from their constitution, whilst substances, such as sugar and alcohol, which do not conduct electricity have no greater osmotic pressure than we should expect. In all probability there is a breaking up in the molecule of the salt, and the fragments are called ions.

I do not intend to speak of the application of electricity to seeds by overhead wires. The electro-chemical treatment of seeds is quite distinctive. Various electrolytes, that is, various solutions, are used; the seed is steeped in the solution, and a current of low-tension electricity is passed for varying times according to the nature of the grain. Now, the dried grain, in whose latent life the currents of absorption and elimination have ceased, is ionised, or electrified.

The first electrolytes used were the chemical manures. Wheat was steeped in a solution of nitrate of soda; electricity of low tension was passed through it. The wheat was sown. The results were satisfactory. The treated grain showed plants which were stronger, taller, and with more and better ears. In all these experiments, along with the treated seed, under exactly similar conditions, untreated seeds were sown. The patient investigator was rewarded, and in course of time the process became a practical success.

The seed is now treated in tanks of varying sizes, to hold ten or twenty sacks. Each end of the tank has an iron plate serving as the electrode. The source of the electricity is the town supply or a dynamo. A rheostat for regulating the strength of the current is, of course, necessary. The seed is treated, the solution run off, the seed taken out and dried. The drying is very important, for if the grain is overdried it will be killed; if underdried, considerable damage is done to germination. The drying may be done in a malt kiln or by any of the various methods.

The seed should be sown within a month or two of the treating. Mr. Baines has shown in his studies on vegetable electro-physiology that every seed and every vegetable has a permanent electrical charge. There can be no question that there is a tendency to revert to the normal if the seed is kept too long. The results in regard to wheat, oats, and barley are unqualified successes. Potatoes treated show a 50 per cent. increase in weight. The electrified seed shows an increase in grain and sometimes in straw. The increased yield of grain is well over 6 bushels per acre.

The Board of Agriculture has investigated the matter; has reaped, thrashed, and weighed samples of the crops, and has found an average increase of 10½ bushels to the acre to the credit of the electrified seed. The Board, however, are not prepared to say to what extent this is due to the chemical soaking or to the electrical change.

The inventor of this process, having succeeded, after years of experimentation, in bringing the process to maturity for the principal farm crops, and in securing a large increase of yield from all kinds of seed corn, is now turning his attention to other agricultural and to horticultural seeds. Much progress has been made in adapting the process to seeds of garden crops as well as to those of farm crops other than cereals; but with respect to them the process is not yet quite sufficiently matured to warrant him in recommending its general adoption.

During the season now closing more than 500 farmers have grown cereals treated by the Wolfryn electrical process. Measured tests have been made by some of the farmers themselves, and some by an expert appointed by the chief executive officer of the Dorset Executive Committee. The Agricultural Committee of Bucks have made an examination of some of the treated crops in their county. A few only of the results have yet been definitely reported.

* Part of a paper read before the Agricultural Section of the British Association, September 9.

